# A rare case of cervical tuberculosis simulating carcinoma cervix: a case report

**DOI:** 10.1186/1757-1626-2-161

**Published:** 2009-10-20

**Authors:** Swati Agrawal, Monika Madan, Nitin Leekha, Chitra Raghunandan

**Affiliations:** 1 Department of Obstetrics & Gynecology, Lady Hardinge Medical College, New Delhi, India

## Abstract

**Background:**

This is an unusual case of a 26-year-old P2L2 lady who presented with chief complaints of pain abdomen and irregular bleeding p/v with history of post-coital bleeding.

**Case report:**

On per speculum examination, cervix was replaced by an irregular friable growth, which was bleeding on touch. A clinical diagnosis of carcinoma cervix was made but the cervical biopsy revealed granulomatous inflammation with presence of acid-fast bacilli on cervical smear consistent with tuberculosis. The patient responded to six months of anti-tubercular therapy.

**Conclusion:**

To conclude, cervical tuberculosis should be considered in the differential diagnosis of carcinoma cervix in young women with suspicious cervix.

## Case report

A case of tuberculosis of the cervix presenting as cervical carcinoma is being reported for its rarity.

A 26-year-old P_2_L_2 _Indian lady, housewife by occupation, presented with chief complaints of pain abdomen, irregular bleeding and discharge per vaginum for three years. She had history of post-coital bleeding and inter-menstrual bleeding; and significant weight loss over the last two years. There was no history of genitourinary malignancy or tuberculosis in the past or in the family. The patient was a non-smoker, non-alcoholic and did not have any other significant medical or surgical illness in the past.

**Figure 1:**
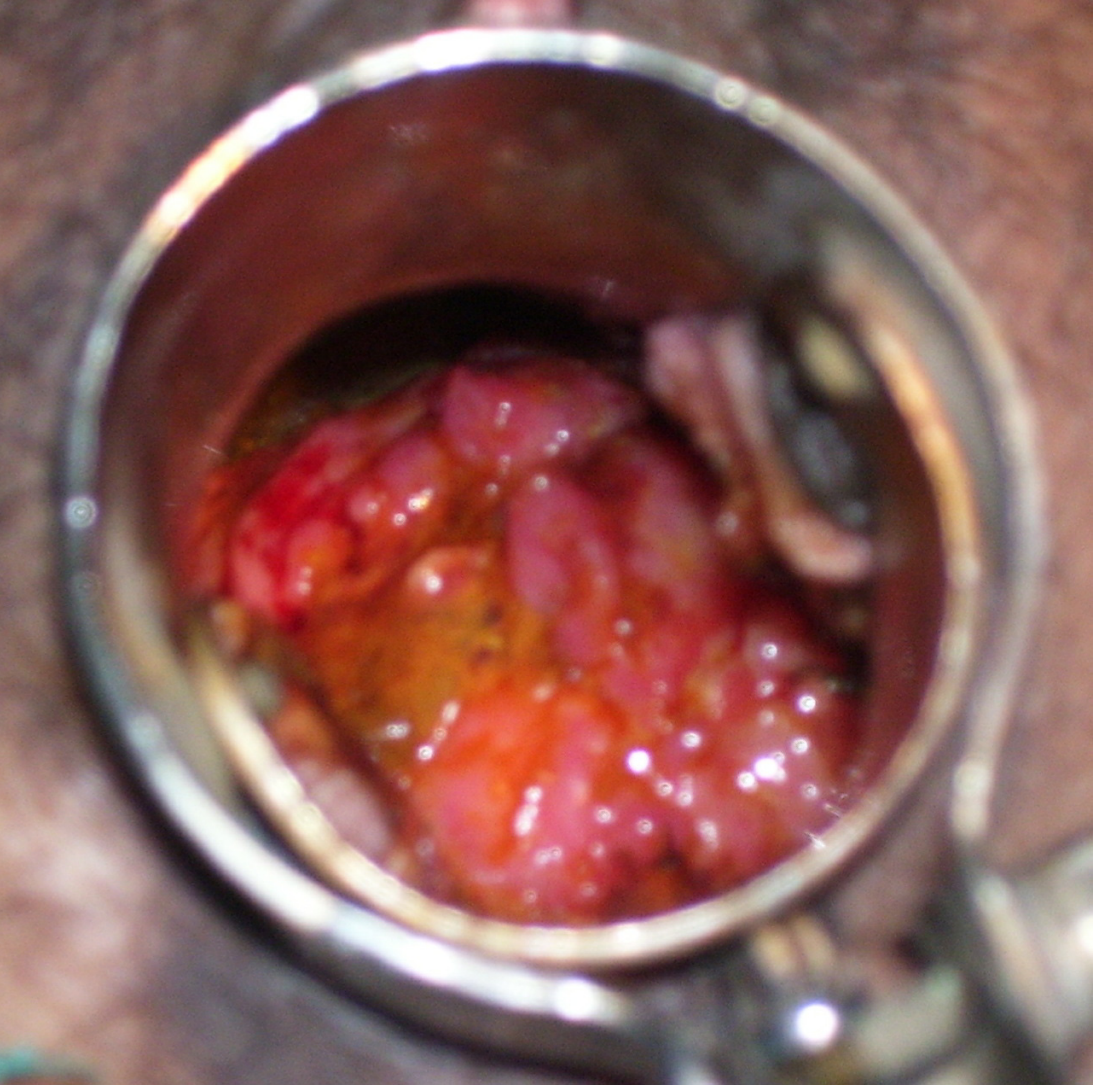
**Per speculum view of the cervix at the time of presentation**.

**Figure 2:**
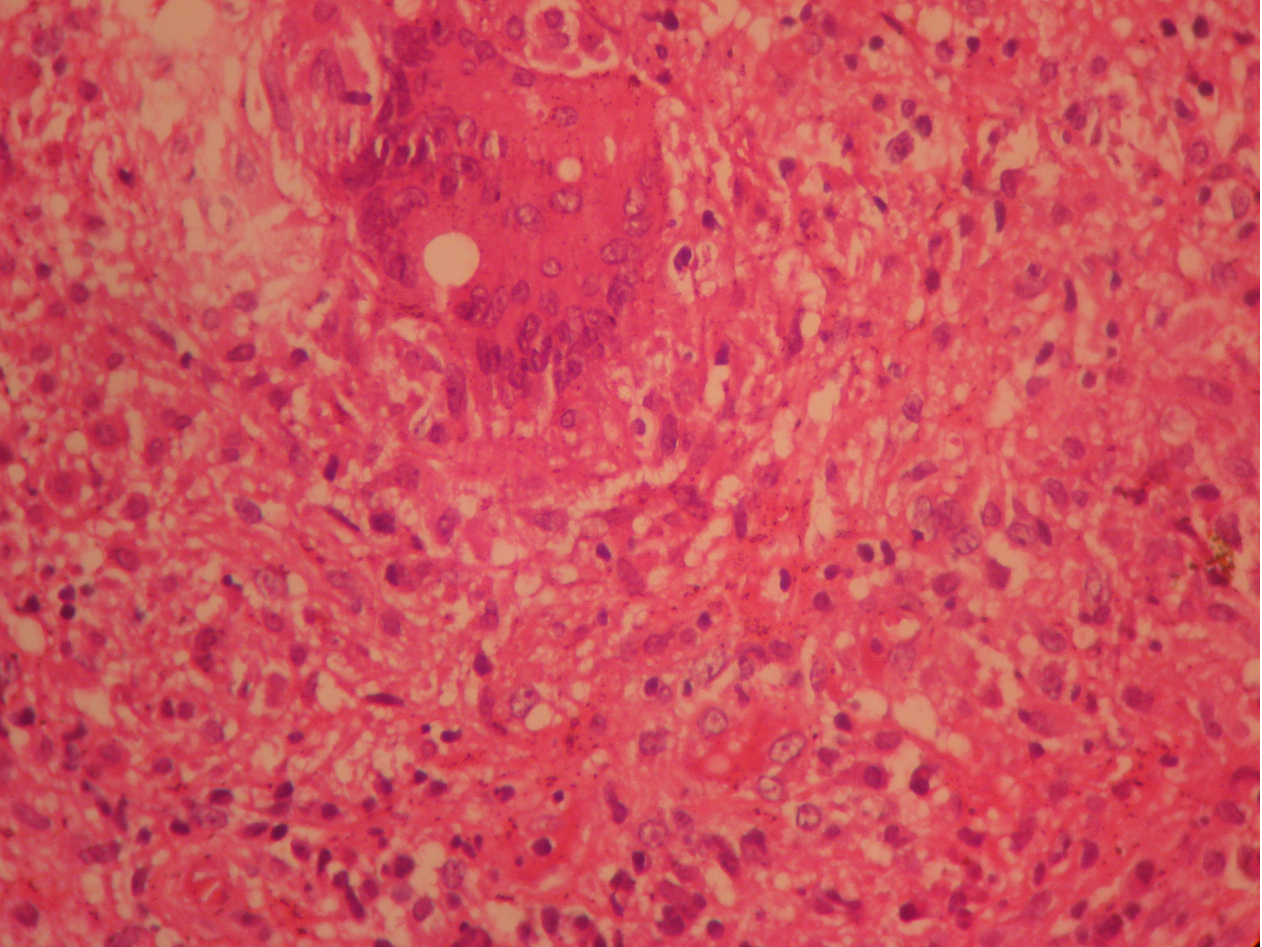
**Histopathological examination of the cervical biopsy showing granulomatous inflammation**.

General physical examination was essentially normal with no palpable lymph nodes. Systemic examination did not reveal any abnormality. On per speculum examination, cervix was replaced by an irregular friable growth, which was bleeding on touch (Figure 1). On bimanual examination, same growth was felt. Uterus was anteverted, normal in size and bilateral fornices were free. Per rectal examination did not reveal any induration or nodularity of parametria and rectal mucosa was smooth and freely mobile. Colposcopic examination showed increased vascularity without any acetowhite or iodine negative areas. PAP smear showed epitheloid like cell clusters without any dysplasia. Biopsy taken from the cervical growth revealed granulomatous inflammation with caseous necrosis. Smear from cervix was found positive for acid-fast bacilli. Endometrial biopsy was normal with no AFB (Figure 2). A chest radiograph was normal. Sputum and urine samples were negative for AFB and failed to culture mycobacterium. CECT abdomen showed bulky cervix with evidence of soft tissue streaking in parametrium. HIV 1 and 2 was negative. Patient was started on antitubercular treatment (four drugs: isoniazid, ethambutol, rifampicin and pyrazinamide) and discharged. At six months, the cervix had an almost normal appearance and there was complete relief from symptoms.

## Discussion

Genital tuberculosis is common in 20-40 years of age group in developing countries. Genital organs most frequently affected include fallopian tubes (95-100%), endometrium (50-60%), and ovaries (20-30%) [1]. Tuberculosis of the cervix is rare and accounts for 0.1-0.65% of all cases of tuberculosis (TB) and 5-24% of genital tract TB [2]. Genital Tuberculosis is a major socioeconomic burden in India, afflicting 14 million people, mostly in the reproductive age group (15-45 years). It is involved in about 5-16%of cases of infertility among Indian women, though the actual incidence may be under-reported due to asymptomatic presentation of genital tuberculosis and paucity of investigations [3-5].

Pelvic organs are infected from a primary focus, usually the chest, by hematogenous spread. The cervix is infected, as a part of this process, by lymphatic spread or by direct extension. In rare cases, cervical TB may be a primary infection, introduced by a partner with tuberculous epididymitis or other genitourinary disease. It has been suggested that sputum, used as a sexual lubricant, may also be a route of transmission [1].

Cervical tuberculosis may present as papillary or vegetative growths on cervix, a military appearance, and/or ulceration simulating invasive cervical cancer [1]. The diagnosis of cervical tuberculosis is usually made by histological examination of the cervical biopsy, which reveals caseating granulomas. Staining for acid-fast bacilli was not found to be very useful. Isolation of the mycobacterium is the gold standard for diagnosis but a third of cases are culture negative. Therefore, the presence of typical granulomata is sufficient for diagnosis if other causes of granulomatous cervicitis are excluded or a primary focus identified. The differential diagnoses for granulomatous disease of the cervix include amoebiasis, schistosomiasis, brucellosis, tularaemia, sarcoidosis, and foreign body reaction[6]. The cervix should respond to six months of standard therapy [7].

This case emphasizes that though uncommon, tuberculosis is an important alternative in the differential diagnosis of a malignant appearing lesion of the cervix. With resurgence of tuberculosis worldwide, there should be a high index of suspicion of tuberculosis in women with an abnormal cervical appearance.

## Abbreviations

P2L2: Para 2 with 2 living issues; PAP smear: Papanicolaou smear; AFB: Acid-fast bacilli

## Consent

Written informed consent was obtained from the patient for publication of this case report and accompanying images. A copy of the written consent is available for review by the Editor-in-Chief of this journal.

## Competing interests

The authors declare that they have no competing interests.

## Authors' contributions

SA examined the patient, made a clinical diagnosis and conducted the cervical biopsy while CR analyzed and interpreted the data. MM performed the colposcopic examination of the cervix, and was a major contributor in writing the manuscript. All authors read and approved the final manuscript.
